# Determinants of chronic otitis media with effusion in preschool children: a case–control study

**DOI:** 10.1186/s12887-016-0767-7

**Published:** 2017-01-06

**Authors:** Rebecca E. Walker, Jim Bartley, David Flint, John M. D. Thompson, Edwin A. Mitchell

**Affiliations:** 1 Department of Paediatrics: Child and Youth Health, The University of Auckland, Private Bag 92019, Auckland, 1142 New Zealand; 2 Division of Otolaryngology-Head and Neck Surgery, Counties-Manukau District Health Board, Manukau SuperClinic™, PO Box 98743, Manukau City, Auckland 2241 New Zealand

**Keywords:** Otitis media with effusion, Risk factors, Upper respiratory tract infections, Nasal obstruction, Snoring, Cow’s milk, Long labour, Biofilms

## Abstract

**Background:**

Chronic otitis media with effusion (COME) is a prevalent upper airway infection resulting in hearing loss. The aim of this research was to determine risk factors for COME in preschool children.

**Methods:**

A case–control design was conducted in Auckland, New Zealand from May 2011 until November 2013. The cases were children aged 3 and 4 years referred for tympanostomy tube placement due to a diagnosis of COME (*n* = 178). The controls were a random sample of healthy children aged 3 and 4 years from primary care practices (*n* = 209). The children’s guardians completed an interviewer-administered questionnaire that covered topics including socio-demographic information, pregnancy and birth, infant feeding practices, home environment, and respiratory health. In addition, skin prick tests for atopy were performed. Odds ratios (OR) estimating the risk of COME independently associated with the exposures were calculated using a logistic regression model.

**Results:**

Children with COME frequently had nasal obstruction (OR: 4.38 [95% CI: 2.37–8.28]), always snored (OR: 3.64 [95% CI: 1.51–9.15]) or often snored (OR: 2.45 [95% CI: 1.04–5.96]), spent more hours per week in daycare (OR per hour/week: 1.03 [95% CI: 1.00–1.05]), had frequent colds (OR: 2.67 [95% CI: 1.59–4.53]), had siblings who had undergone tympanostomy tube placement (OR: 2.68 [95% CI: 1.22–6.02]), underwent long labour (OR: 2.59 [95% CI: 1.03–6.79]), and had early introduction of cow’s milk (OR: 1.76 [95% CI: 1.05–2.97]). Asian ethnicity (OR: 0.20 [95% CI: 0.07–0.53]) and having older siblings (OR: 0.54 [95% CI: 0.31–0.93]) were inversely associated with COME.

**Conclusion:**

COME in preschool children was associated with pathogen exposure, respiratory infection, and nasal obstruction. Strategies to prevent pathogen transmission warrant investigation. The novel findings of long labour and early cow’s milk introduction require replication in future studies.

## Background

Otitis media with effusion (OME) is a childhood condition where fluid gathers behind the tympanic membrane. OME is a common reason for doctors’ visits, antibiotic use and surgery [1]. Chronic otitis media with effusion (COME), defined as effusion lasting 3 or more months, causes hearing loss that may lead to learning delays and behavioural problems [2].

COME is caused by bacteria and viruses entering the middle ear from the upper respiratory tract, leading to a chronic inflammatory response. COME chronicity may be explained by the formation of biofilms in the middle ear [3]. Biofilms are sessile communities of microorganisms that can evade the host’s immune system and are often resistant to antibiotic treatment [1]. Risk factors associated with COME include young age, ethnicity, family history, breastfeeding practices, exposure to other children, and upper respiratory infection (URI) [4]. Acute otitis media (AOM) and OME often follow each other, and can be viewed as aspects of a disease continuum.

While many risk factors for COME have been reported, there is a lack of recent research in which a wide range of potential determinants and confounders are considered together. The aim of the study was to examine a comprehensive array of variables, including prenatal and perinatal factors for which research is lacking [4], to identify risk factors that are independently associated with COME.

## Methods

### Study design

A case–control study of children aged 3 and 4 years was conducted, to compare children with COME to healthy children.

### Setting

Recruitment and data collection were conducted between May 2011 and November 2013. Subjects were assessed in interview rooms at Waitakere Hospital or North Shore Hospital, in the Waitemata District Health Board (WDHB) catchment area in Auckland. WDHB is the largest of 20 district health boards in New Zealand (NZ), with a population of approximately 560,000 people, and the third most affluent. Its population consists of 10% Maori, 7% Pacific Island, and 19% Asian people, with the remaining 64% being European or Other ethnicities [5]. Children in NZ have free hospital care and primary care practice visits.

### Participants

Cases were children aged 3 or 4 years referred for tympanostomy tube placement (TTP) at Waitakere Hospital, who had a recent medical history of COME and/or signs of COME confirmed by an otorhinolaryngologist during surgery.

Controls were selected at random from children enrolled in primary care practices in proportion to the number and sex of children those practices had referred to Waitakere Hospital for TTP in 2010. This approach was designed to recruit controls from the same population that cases were referred from, to mitigate against selection bias. Eligible controls were aged 3 or 4 years, had no medical history of TTP, no episodes of OME lasting longer than one month in the past year, and never had OME lasting longer than three months.

Cases and controls were excluded if they had craniofacial abnormalities including Down syndrome or cleft palate, or immunodeficiency, as risk factors for COME in children with these conditions may not be generalizable.

### Variables

An interviewer administered a questionnaire to obtain information regarding socio-demographic factors, pregnancy, feeding practices, allergy, nasal symptoms, and childcare environment. The interviewer was blind to whether the participant was a case or control. Recall misclassification and bias was mitigated by using a standardized questionnaire including validated questions where available.

Respondents could provide multiple ethnicities, which were coded as a single ethnicity using the “prioritized output” method from the NZ Ministry of Health ethnicity data protocol [6]. The ethnicity categories were Maori, Pacific Island, Asian, European, and Other. European and Other ethnicity were grouped into a single European/Other category for analysis due to the small number of subjects of Other ethnicity. Residential address was used to assign NZ Deprivation Index (NZDep) ratings. NZDep is a validated proxy measure of socio-economic status (SES) ranging from 1 (least deprived) to 10 (most deprived) [7]. Crowding was assessed by number of people per bedroom. Long labour was defined as total length of labour (stage 1 and 2) lasting for 21 h or longer in first-born children or otherwise lasting 14 or more hours. These cutoffs were based on the top 5^th^ percentile of labour length in our control subjects. Mode of delivery was categorized as unassisted (normal vaginal delivery), elective Caesarean section, emergency Caesarean section, or forceps/vacuum delivery. Reflux in infancy was defined as bringing up milk and crying in pain after feeding. Colic in infancy was defined as prolonged crying, reddened face, tight body, and bringing up knees. Time of first exposure to cow’s milk (not including formula or foods containing cow’s milk) was dichotomized around the median time in the controls, which was 13 months. Allergic symptoms were assessed using questions from the ISAAC questionnaire (http://isaac.auckland.ac.nz). Allergic rhinoconjunctivitis was defined as sneezing, or a runny, or blocked nose, combined with itchy-watery eyes, in the absence of a cold or flu.

The child’s height and weight were measured, their temperature taken using an ear thermometer, pneumatic otoscopy and tympanometry were performed, and skin prick tests for house dust mite, cat pelt, dog, mold, birch tree and grass mix were administered following the Australasian Society of Clinical Immunology and Allergy protocol [8].

Other factors analyzed included maternal age, smoking during pregnancy, induced labour, small for gestational age (bottom 10% of birth weight adjusted for gestational age, parity, sex, and ethnicity) [9], season of birth, pacifier use, age of solid food introduction, maternal and child supplement use, and body mass index (BMI) Z-score (adjusted for age and sex) [10].

### Statistical methods

To detect an odds ratio of 2 at the 5% level and 80% power, assuming an exposure frequency of at least 20% in the controls, a sample size of 173 cases and 173 controls is required.

Statistical analysis was conducted using JMP 12, SAS Institute Inc., Cary, NC, 1989–2015. Categorical variables were analyzed using a chi-squared test and continuous variables were analyzed using a Student *t*-test. Variables with a *P* value ≤ .1 were entered into a logistic regression model. Age in months, the child’s sex, and NZDep were retained in the model as potential confounders. The model was reduced by excluding the least significant variable with *P* > .05, in a stepwise manner until all variables in the model remained significant at the 5% level. This was done to maximize sample size, due to missing data for some variables. Sensitivity analyses were conducted by adding each removed variable back into the final model to ensure that they remained non-significant and did not impact the variables in the model.

## Results

**Fig. 1:**
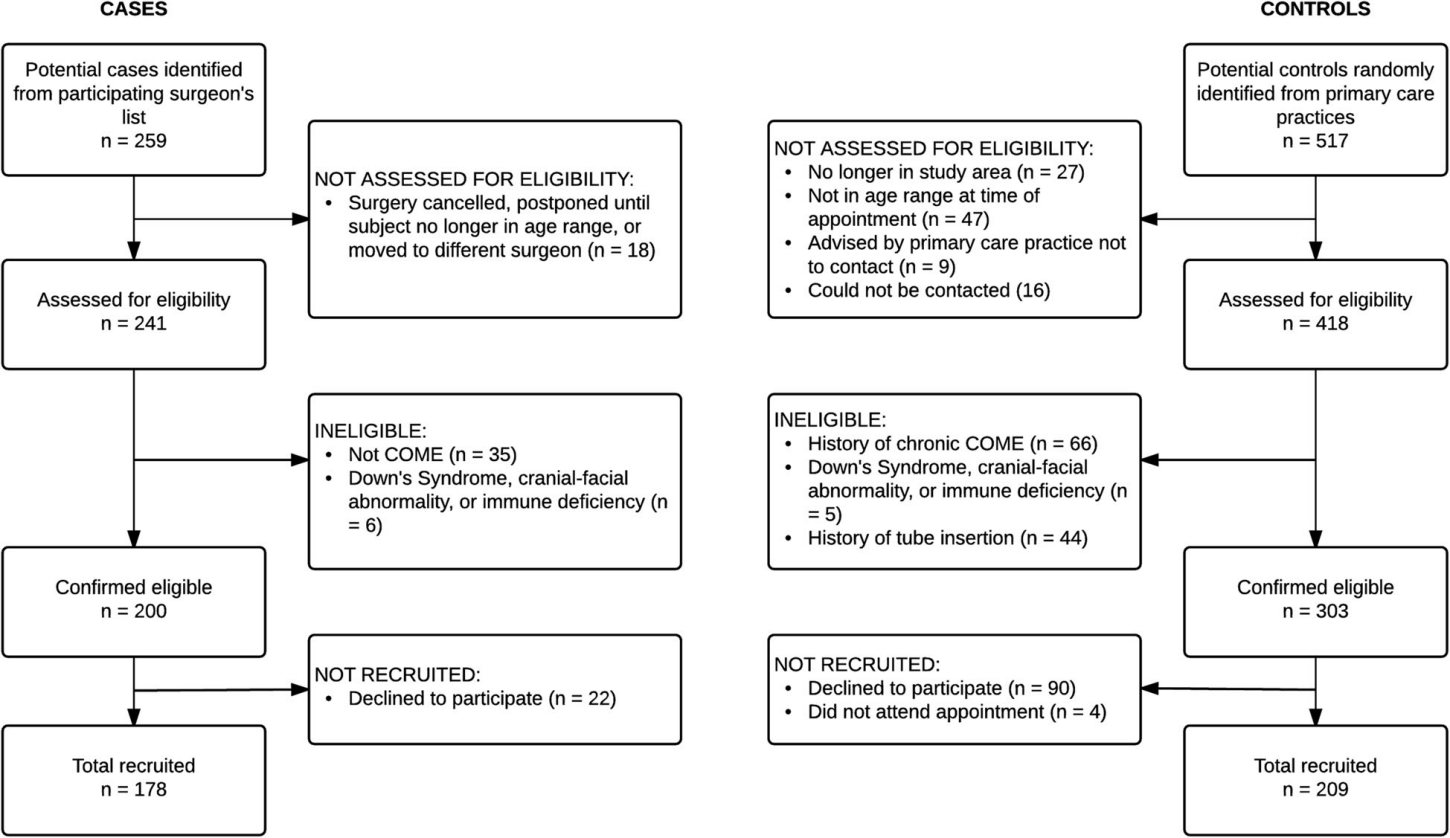
Recruitment stages and participation

Of 259 potential cases identified, 18 were excluded due to surgery being cancelled or postponed, 35 due to not having COME, and 6 due to Down syndrome, cranial-facial abnormalities, or immunodeficiency. Of 200 cases confirmed eligible, 178 (89%) were enrolled. Of 517 potential controls identified, 27 were no longer in the study area, 47 were no longer in the age range, and 5 had Down syndrome, cranial-facial abnormalities or immunodeficiencies. A history of COME was found in 66 potential controls, 44 had TTP, 16 could not be contacted, and we were advised not to contact 9 subjects by their primary care practices. Of 303 controls confirmed eligible, 209 (69%) were enrolled (Fig. 1).

The mean age of the cases was 47.8 months [standard deviation (SD) = 6.79], significantly younger than the controls who had a mean age of 49.3 months [SD = 6.67] (*P* = .04). Of the cases, 62% were male compared to 58% of controls (*P* = .42). Mean NZDep in the cases was 5.5 [SD = 2.66], higher (i.e. more deprived) than the controls who had a mean of 4.8 [SD = 2.65] (*P* = .01).

Variables with *P* values over .1 univariably that were not analyzed further were: induced labour, small for gestational age, season of birth, pacifier use, age of solid food introduction, maternal and child supplement use, positive skin prick test, eczema, BMI, and crowding.

Upright bottle-feeding was excluded from further analysis because children with COME are recommended to be bottle-fed upright in NZ [11]. Age at first episode of AOM and age of first starting antibiotics were excluded because AOM is part of the same disease continuum as COME and antibiotics are frequently prescribed for children with OM.

Variables with *P* values ≤ .1 univariably (Table 1) were analyzed using logistic regression. Those with *P* values over .05 in the stepwise model that were not entered into the final model were: maternal smoking during pregnancy, mode of delivery, breastfeeding duration, formula feeding, colic, reflux, parental smoking, childhood use of vitamin C, mouth breathing, full vaccination, probiotic use, wheezing, allergic rhinoconjunctivitis, runny nose, and maternal history of OM.

**Table 1:** Variables with *P* value of .1 or under in univariable analysis

Variable	COME *n* (%)	Controls *n* (%)	OR (CI)	*P* value
Ethnicity				<.001
European/Other	102 (57.3)	112 (53.6)	1.00	
Asian	7 (3.9)	33 (15.8)	0.23 (0.09, 0.52)	
Maori	47 (26.4)	38 (18.2)	1.36 (0.82, 2.26)	
Pacific Island	22 (12.4)	26 (12.4)	0.93 (0.49, 1.74)	
Smoking				.006
Neither smoke	109 (61.2)	161 (77)	1.00	
Mother smokes	15 (8.4)	12 (5.7)	1.05 (0.83, 4.17)	
Father smokes	19 (10.7)	16 (7.7)	1.75 (0.86, 3.60)	
Both smoke	35 (19.7)	20 (9.6)	2.58 (1.43, 4.78)	
Long labour				.01
No	153 (87.4)	198 (94.7)	1.00	
Yes	22 (12.6)	11 (5.2)	2.59 (1.22, 5.50)	
Maternal smoking during pregnancy				.07
No	136 (76.4)	174 (83.7)	1.00	
Yes	42 (23.6)	34 (16.3)	1.58 (0.95, 2.62)	
Mode of delivery				.06
Unassisted	102 (57.3)	128 (61.2)	1.00	
C sect – elective	15 (8.4)	31 (14.8)	0.61 (0.31, 1.18)	
C sect – emergency	38 (21.3)	30 (14.4)	1.61 (0.93, 2.78)	
Forceps or vacuum	23 (12.9)	20 (9.6)	1.46 (0.76, 2.82)	
Breastfed duration (months)	178	208	0.96 (0.93, 0.99)	.003
Formula				.006
No	29 (16.3)	58 (27.9)	1.00	
Yes	149 (83.7)	150 (72.1)	1.99 (1.20, 3.28)	
Cow’s milk start < 13 months of age				.009
No	71 (39.9)	111 (53.1)	1.00	
Yes	107 (60.1)	98 (46.9)	1.71 (1.14, 2.56)	
Colic				<.001
Never	54 (30.3)	88 (42.3)	1.00	
Seldom	67 (37.6)	77 (37)	1.42 (0.89, 2.28)	
Often	21 (11.8)	28 (13.5)	1.22 (0.63, 2.36)	
Always	36 (20.2)	15 (7.2)	3.91 (1.99, 7.99)	
Reflux				.005
Never	110 (61.8)	150 (72.1)	1.00	
Seldom	31 (17.4)	39 (18.8)	1.08 (0.63, 1.84)	
Always and often	37 (20.8)	19 (9.1)	2.66 (1.47, 4.95)	
Probiotic use in last 12 months				.04
No	153 (86.4)	193 (92.8)	1.00	
Yes	24 (13.6)	15 (7.2)	2.02 (1.02, 3.98)	
Vitamin C use in last year				.07
No	137 (77.4)	144 (69.2)	1.00	
Yes	40 (22.6)	64 (30.8)	0.66 (0.42, 1.04)	
Allergic rhinoconjunctivitis in last 12 months				.01
No	148 (83.1)	191 (91.4)	1.00	
Yes	30 (16.9)	18 (8.6)	2.15 (1.15, 4.01)	
Wheeze in last 12 months				.007
No	99 (55.6)	144 (68.9)	1.00	
Yes	79 (44.4)	65 (31.1)	1.77 (1.17, 2.68)	
Blocked nose				<.001
Only with cold, rarely or never	84 (47.1)	180 (86.1)	1.00	
Always or often	94 (52.8)	29 (13.9)	6.95 (4.25, 11.34)	
Variable	COME *n* (%)	Controls *n* (%)	OR (CI)	*P* value
Snoring				<.001
Never	14 (7.9)	49 (23.6)	1.00	
Seldom	29 (16.3)	58 (27.9)	1.75 (0.84, 3.76)	
Only with cold	21 (11.8)	38 (18.3)	1.93 (0.88, 4.37)	
Often	46 (25.8)	37 (17.8)	4.35 (2.13, 9.32)	
Always	68 (38.2)	26 (12.5)	9.15 (4.44, 19.89)	
Runny nose				<.001
No	11 (6.2)	35 (16.7)	1.00	
Only with cold	85 (47.8)	126 (60.3)	2.15 (1.06, 4.64)	
Yes – clear	51 (28.7)	36 (17.2)	4.50 (2.08, 10.40)	
Yes – purulent	31 (17.4)	12 (5.7)	8.20 (3.28, 22.15)	
Mouth breathing				<.001
Never	19 (10.7)	63 (30.4)	1.00	
Rarely	39 (22.0)	44 (21.3)	2.94 (1.52, 5.83)	
Only with cold	28 (15.8)	52 (25.1)	1.79 (0.90, 3.59)	
Often	50 (28.2)	38 (18.4)	4.36 (2.28, 8.63)	
Always	41 (23.2)	10 (4.8)	13.59 (5.95, 33.63)	
4 or more colds in last 12 months				<.001
No	61 (34.3)	145 (69.4)	1.00	
Yes	117 (65.7)	64 (30.6)	4.35 (2.84, 6.66)	
Sibling tympanostomy tubes				.002
No	141 (79.2)	189 (90.4)	1.00	
Yes	37 (20.8)	20 (9.6)	2.48 (1.38, 4.46)	
Daycare hours per week	174	208	1.02 (1.00, 1.04)	.03
Older siblings				.07
No	82 (46.1)	77 (36.8)	1.00	
Yes	96 (53.9)	132 (63.2)	0.68 (0.45, 1.03)	
Maternal OM				.02
No	116 (65.5)	156 (76.0)	1.00	
Yes	61 (34.5)	49 (23.9)	1.67 (1.07, 2.62)	
Fully vaccinated				.10
No	16 (9)	30 (14.4)	1.00	
Yes	162 (91)	178 (85.6)	1.71 (0.90, 3.25)	

In the final multivariable model (Table 2), Asian ethnicity was inversely associated with COME as compared to European/Other ethnicity (OR: 0.20 [95% CI: 0.07–0.53]). Deprivation was not associated. Long labour (OR: 2.59 [95% CI: 1.03–6.79]), starting cow’s milk before 13 months of age (OR: 1.76 [95% CI: 1.05–2.97]), and having a blocked nose (always or often, OR: 4.38 [95% CI: 2.37–8.28]) were significant risk factors. Snoring was associated with COME when compared to never snoring (always, OR: 3.64 [95% CI: 1.51–9.15]) or often (OR: 2.45 [95% CI: 1.04–5.96]) Four or more colds in the last 12 months (OR: 2.67 [95% CI: 1.59–4.53]), siblings with a history of TTP (OR: 2.68 [95% CI: 1.22–6.02]), and number of hours per week at daycare (OR per hour: 1.03 [95% CI: 1.00–1.05]) were all risk factors, and having older siblings was inversely associated (OR: 0.54 [95% CI: 0.31–0.93]).

**Table 2:** Risk factors for COME in final multivariable model

Variable	Adjusted OR (CI)	*P* value
Age (per month)	0.96 (0.92, 1.00)	.04
Sex		.53
Female	1.00	
Male	1.18 (0.70, 1.99)	
NZDep (per unit)	1.06 (0.96, 1.17)	.27
Ethnicity		.005
European/Other	1.00	
Asian	0.20 (0.07, 0.53)	
Maori	0.90 (0.47, 1.72)	
Pacific Island	1.33 (0.58, 3.04)	
Long labour		.04
No	1.00	
Yes	2.59 (1.03, 6.79)	
Cow’s milk start < 13 months of age		.03
No	1.00	
Yes	1.76 (1.05, 2.97)	
Blocked nose in last 12 months		<.001
Only with cold, rarely and never	1.00	
Always and often	4.38 (2.37, 8.28)	
Snoring in last 3 months		.02
Never	1.00	
Seldom	1.29 (0.55, 3.10)	
Only with cold	1.51 (0.59, 3.94)	
Often	2.45 (1.04, 5.96)	
Always	3.64 (1.51, 9.15)	
4 or more colds in last 12 months		<.001
No	1.00	
Yes	2.67 (1.59, 4.53)	
Sibling tympanostomy tubes		.01
No	1.00	
Yes	2.68 (1.22, 6.02)	
Daycare hours per week (per hour)	1.03 (1.00, 1.05)	.02
Older siblings		.03
No	1.00	
Yes	0.54 (0.31, 0.93)	

## Discussion

To address the many inter-related factors associated with COME, we included a comprehensive set of variables in our regression analysis. In addition to well known risk factors such as frequent URI, daycare attendance, and sibling history of TTP, we also identified some respiratory risk factors that were independently associated with COME, notably nasal obstruction and snoring. Prenatal and perinatal events also appear to impact the risk of COME in preschoolers, including long labour and early introduction of cow’s milk.

URI is a frequently observed risk factor [4, 12]. In animal models, bacteria in the nose are more likely to ascend to the middle ear if respiratory viruses are also present [13]. Our finding regarding URI may reflect viruses acting as a compounding risk factor through polymicrobial interactions with bacteria in the nose and nasopharynx.

Daycare attendance exposes children to infection that can lead to COME [12]. Children congregating at daycare increases the transfer of viruses, and the transmission of bacteria that may survive in a biofilm state in mucous secretions on toys and other surfaces [14]. Sibling TTP being a risk factor may reflect shared family environment including parental healthcare practices, awareness of COME, or exposure to the same pathogens. It may also indicate familial predisposition, which is a risk factor supported by a number of twin studies [15], however a history of maternal OM was not significant in the multivariable analysis.

Reports as to whether atopy and allergic rhinitis are risk factors for OME are mixed [4]. We did not find atopic diseases to be risk factors for COME. The discrepancy among studies could be explained by blocked nose or URI acting as confounders, as these conditions are associated with both allergic conditions and COME. We used a validated questionnaire for rhinoconjunctivitis, eczema and asthma, which may have improved our specificity in identifying allergic diseases.

Frequent nasal obstruction in the last 12 months was reported in 53% of cases compared to 14% of controls, while frequent snoring in the last 3 months was found in 64% of cases versus 30% of controls. Nasal obstruction has been considered to be related to COME only via its associations with allergy and URI [16], however there is also evidence to support our finding that nasal obstruction and snoring can be associated with OM independently of allergy and colds [17–19]. Our observation that nasal obstruction was associated with COME independently of rhinitis, atopy, and URI raises the question of what other mechanism could be underlying this relationship. Given the importance of bacterial biofilms in the middle ear in causing COME [3], a possible explanation is that biofilms in the nose and nasopharynx act as a common factor for both chronic nasal obstruction and COME. In animal models biofilms can spread from the nose to the middle ear, leading to COME [13]. Nasal biofilms are associated with increased nasal resistance, a measure of nasal obstruction [20]. The otopathogen non-typable *Haemophilus influenza* requires anaerobic conditions to form biofilms [21], and may therefore be aided by nasal congestion. Although direct evidence that nasal and nasopharyngeal biofilms connect nasal obstruction to COME is lacking, adenoid hypertrophy in particular is a risk factor for nasal obstruction, snoring, and COME, with the preferred explanation being that adenoids can act as a reservoir for otopathogens [22, 23].

In addition to respiratory factors, we made several findings regarding socio-demographic factors, perinatal factors and feeding practices. Maori and Pacific Island children in NZ are more likely to fail preschool hearing tests [24]. The prevalence of OME in 2 year old Pacific Island children has been measured at 25.4%, however chronicity and other ethnicities were not studied, and subjects were recruited from a less affluent area of Auckland [18]. We did not observe Maori or Pacific Island ethnicities to be risk factors for COME. If a correlation exists, it may be concealed by these families being less likely to attend primary care practices for COME, as our subjects are limited to those referred for surgery.

Asian ethnicity was inversely associated with COME. It is also inversely correlated with a diagnosis of AOM in the US [25]. A longitudinal study could determine whether this reflects a lower likelihood of referral/attendance for TTP surgery, genetic propensity, or cultural differences such as diet, housing, or hygiene practices.

Low SES is sometimes linked to OM [4]. In NZ, deprivation is associated with infectious diseases [26], surgical interventions for OM [27], and infection with the otopathogen *Staphylococcus aureus* [28]. However, we did not find deprivation to be associated with COME. Crowding and tobacco smoke exposure were also not identified as risk factors, in contrast to some previous studies [29, 30]. It appears that the presence of COME is better predicted by other potentially related factors in our subjects.

Having older siblings was inversely correlated with COME. Previous research has found this to be a risk factor that declines with age, no longer being associated by 3 years of age [12]. Exposure to commensal bacteria from older siblings may help to protect children from infections and allergic manifestations [31].

Long labour was associated with COME. Prolongation of labour is the most common cause of maternal fever during labour, and is also associated with instrument use, emergency Caesarean section deliveries, and admission to neonatal care units [32–34]. In cases of maternal fever, prophylactic antibiotics are often administered to mother and newborn, which may affect the infant’s microbiome and thereby decrease their resistance to colonization with pathogens [31, 34].

Longer duration of breastfeeding is sometimes reported as a protective factor against OM, and early introduction of infant formula or cow’s milk is sometimes found to be a risk factor [35]. The introduction of cow’s milk before 13 months of age was a risk factor for COME. Cow’s milk is not recommended until after 12 months of age, because its composition is significantly different to breast milk and infant formula, and its use can lead to iron deficiency [36, 37]. Another consideration is cow’s milk protein allergy, which is associated with TTP [38]. We did not find infant formula to be a risk factor, even though it usually contains cow’s milk. This could be due to the differences in its content, or the use of hypoallergenic and dairy-free varieties. Infant consumption of cow’s milk also supports a different microbiome composition [39], which could promote pathogen colonization leading to COME.

A recent systematic review concluded that children with COME have a high prevalence of reflux, and suggested that aspiration of pepsin into the airways may result in an inflammatory response in the middle ear cavity [40]. Frequent reflux and colic were not significant in our final model and may only be related to COME via other risk factors.

Study limitations need to be considered. The cases were referred for surgery, so they may not represent all preschool children with COME. Retrospective case–control designs have a potential for selection bias and recall bias. Selection bias was mitigated by recruiting controls from practices that had recently referred preschoolers for surgery and by having a high participation rate. Differential recall bias was mitigated with the use of a standardized questionnaire using validated questions where available. While NZDep is a useful indicator of deprivation, it reflects data from a small area of houses rather than specific households. A major strength of the study is that an otorhinolaryngologist confirmed disease presence at surgery, which is preferable to relying on medical history or tympanometry alone. Furthermore, a comprehensive range of risk factors were explored and the sample size was relatively large, allowing us to have confidence in these findings.

## Conclusion

Our results support well-established risk factors for COME relating to exposure to infection, specifically URI, daycare attendance, and sibling TTP.

There is growing interest in how respiratory microbiota may support the immune system against conditions such as COME. More investigation is required to confirm whether our results regarding older siblings, prolonged labour (with associated antibiotic use), and the timing of cow’s milk introduction reflect effects on the commensal microbiota.

We postulate that biofilms in the nose and nasopharynx link nasal obstruction, snoring, and COME. The child’s nose or nasopharynx is first colonized by otopathogenic bacteria that form biofilms. The resulting chronic inflammation may cause nasal obstruction and snoring, but fails to fully suppress the biofilm infection. The otopathogens pass up the Eustachian tube to the middle ear, a risk that is compounded by viral infection, and form biofilms again in the middle ear. This may produce chronic inflammation in the middle ear, i.e. COME. Research on the association between bacterial biofilms in the nose and nasopharynx, nasal obstruction, and COME is required to test this hypothesis.

## Abbreviations

AOM: Acute otitis media
BMI: Body mass index
COME: Chronic otitis media with effusion
ISAAC: The International Study of Asthma and Allergy in Children
NZ: New Zealand
NZDep: New Zealand Deprivation Index
OM: Otitis media
OME: Otitis media with effusion
SD: Standard deviation
TTP: Tympanostomy tube placement
URI: Upper respiratory infection
